# Sampling the structure and chemical order in assemblies of ferromagnetic nanoparticles by nuclear magnetic resonance

**DOI:** 10.1038/ncomms11532

**Published:** 2016-05-09

**Authors:** Yuefeng Liu, Jingjie Luo, Yooleemi Shin, Simona Moldovan, Ovidiu Ersen, Anne Hébraud, Guy Schlatter, Cuong Pham-Huu, Christian Meny

**Affiliations:** 1 Institut de Chimie et Procédés pour l'Energie, l'Environnement et la Santé (ICPEES), UMR 7515 CNRS-University of Strasbourg, ECPM, 25, rue Becquerel, 67087 Strasbourg 02, France; 2 Institut de Physique et Chimie des Matériaux de Strasbourg (IPCMS), UMR 7504 CNRS-University of Strasbourg, 23, rue du Loess, 67034 Strasbourg 02, France; 3 Department of Physics, CNRS-Ewha International Research Center, Ewha Womans University, Seoul 120-750, South Korea

## Abstract

Assemblies of nanoparticles are studied in many research fields from physics to medicine. However, as it is often difficult to produce mono-dispersed particles, investigating the key parameters enhancing their efficiency is blurred by wide size distributions. Indeed, near-field methods analyse a part of the sample that might not be representative of the full size distribution and macroscopic methods give average information including all particle sizes. Here, we introduce temperature differential ferromagnetic nuclear resonance spectra that allow sampling the crystallographic structure, the chemical composition and the chemical order of non-interacting ferromagnetic nanoparticles for specific size ranges within their size distribution. The method is applied to cobalt nanoparticles for catalysis and allows extracting the size effect from the crystallographic structure effect on their catalytic activity. It also allows sampling of the chemical composition and chemical order within the size distribution of alloyed nanoparticles and can thus be useful in many research fields.

## Abstract (web summary)

As nanoparticles possess technological applications from catalysis to medical therapies, methods to probe their structural properties are crucial. Here, the authors extend a nuclear magnetic resonance method to extract such properties for specific size ranges of noninteracting magnetic particles.

## Graphical abstract

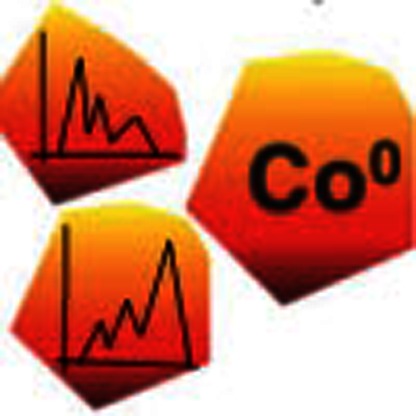

Nuclear magnetic resonance (NMR) is commonly used in chemistry or biology^1^ but is much less popular for studying ferromagnetic systems. However, recent developments^2^,^3^ have shown that NMR can give unique information on the structure, interface morphology and magnetic properties of magnetic thin films^4^,^5^,^6^,^7^,^8^,^9^,^10^,^11^,^12^,^13^,^14^, multilayers^15^,^16^,^17^,^18^,^19^,^20^ or nano-clusters^21^,^22^,^23^,^24^,^25^,^26^. When used for studying ferromagnetic samples, NMR is also called zero field NMR or ferromagnetic nuclear resonance (FNR).

Assemblies of nanoparticles are studied in many research fields such as spintronics, chemistry^21^,^22^,^23^,^24^,^25^,^26^, biology or medicine^27^. Their properties depend on their size, shape, crystallographic structure and chemical composition. Moreover, assemblies of nanoparticles often show wide size distributions, making it difficult to interpret their properties as their structure, composition and chemical order are likely to vary with the size of the particles. Assemblies of nanoparticles are usually studied by techniques, such as X-ray diffraction, transmission electron microscopy (TEM)^27^, atomic probe tomography^28^ or electron holography^29^. However, these techniques present gaps in their application. Indeed, X-ray diffraction requires a minimum diffraction volume and therefore the results are biased towards large particle sizes. High-resolution TEM analysis and other local probe techniques often require specific sample preparations. In addition, the investigated visual field is limited to a small part of the analysed sample (tens of particles), which is not always representative of the sample characteristics.

Compared with the investigation methods discussed in the previous paragraph, FNR methods do not require specific sample preparation (often, even ready-to-use industrial samples can be analysed) and provides information at the opposite scale of the one provided by local techniques. Indeed, FNR allows analysing sample quantities representative of the studied samples: up to 10 mg of Co, typically 10^16^ Co particles. FNR is therefore complementary to the local investigation techniques. In addition, it is especially efficient when wide size distributions cannot be avoided because of the elaboration process. Finally, FNR also allows analysis of the samples in their real macroscopic shapes without tedious sample preparation or time-consuming statistical analysis. FNR also presents drawbacks. Indeed, recent FNR studies^2^,^3^,^30^,^31^ have shown that the interpretation of the properties of ferromagnetic nanoparticles is complex because the FNR line positions depend on the magnetic state of the particles, that is, single domain or multi-domain. In the magnetic single-domain state, the presence of an additional magnetostatic field results in a shift of the resonance lines compared with the regular multi-domain line positions^3^. If both single-domain and multi-domain particles co-exist in the same sample, the two sets of line are superimposed.

Here, we propose the concept of temperature differential FNR (TDFNR) spectra, which allows evaluation of the number of atoms involved in specific size ranges (and the resulting number of particles) within the size distribution of non-interacting ferromagnetic nanoparticles and, at the same time, allows the sampling of the crystallographic structure and chemical composition as a function of the size of the nanoparticles. The technique simultaneously allows us to obtain both sets of information on macroscopic statistically representative samples. This methodology is first applied to the study of supported cobalt nanoparticles used for the Fischer–Tropsch (FT) reaction in the gas-to-liquid process for synthetic fuel production. The results we have obtained shed a new light on the gas-to-liquid research field and might have a significant impact on the development of these processes. In the second example, the method is applied to the sampling of the chemical composition and the chemical order of Co-Fe alloyed nanoparticles. This shows that the method can have a wide field of applications spreading far beyond the fields investigated in the present work.

## Results

### TDFNR spectra

**Figure 1:**
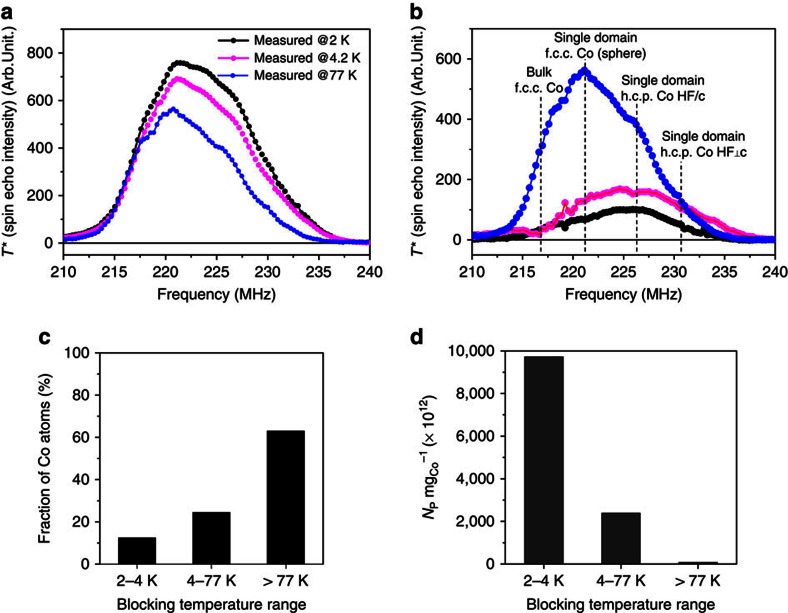
^59^Co FNR spectra of cobalt nanoparticles versus temperature. (**a**) The ^59^Co zero field FNR spectra recorded at 2 (black), 4.2 (magenta) and 77 K (blue). (**b**) TDFNR spectra: in black spectrum measured at 2 K minus spectrum measured at 4.2 K; in magenta spectrum at 4.2 K minus spectrum at 77 K; in blue spectrum at 77 K. Co can have an f.c.c. or an h.c.p. structure and be in two magnetic states: single domain or multi-domain. (**c**) The fractions of cobalt atoms engaged in the different blocking temperature ranges. (**d**) Numbers of Co particles per mg of cobalt (*N*_p_* *mg_Co_^−1^) in different blocking temperature ranges. 2–4.2 K range corresponds to 3–4 nm particles, 4.2–77 K range to 4–10 nm; >77 K corresponds to particles larger than 10 nm.

Our methodology consists in measuring the FNR spectra of the same sample under different temperatures. This is not usually performed in FNR because the signal scales with the inverse of the measurement temperature (1/*T*; nuclear spins are paramagnets), and because measuring at high temperature might lead to a loss of information. In this work, we make use of the loss of information in order to select the size of the investigated particles. The regular 1/*T* temperature dependence of the FNR signal was taken into account in all spectra shown in this paper. Therefore, the changes in shapes and intensities observed as a function of the temperature are related only to the modification of the physical properties of the samples. When performing FNR in ferromagnets, an FNR signal can only be obtained if the magnetization direction of the probed sample is fixed (blocked) during the FNR measurement time scale. In the case of a nanoparticle, for a given volume, the magnetization is not blocked anymore above a specific temperature (blocking temperature)^32^. Such particle is called superparamagnetic and its contribution vanishes from the FNR signal (see the Methods for details). When the FNR measurement temperature is increased, the size limit below which the Co particles become superparamagnetic increases also. It is therefore possible to select the size of the measured particles through the FNR measurement temperature: the average size of the probed particles increases with the increase of the FNR measurement temperature. This is exemplified in the Fig. 1a showing typical FNR spectra (Sample CoDTiS, discussed in details in the next paragraph). These spectra represent a number of atoms versus the radiofrequency field frequency (see the Methods for details). It can clearly be seen that the shapes of FNR spectra does change with the increase of the measurement temperature. A closer look shows that while the FNR intensity close to 217 MHz does stay unchanged for all measurement temperatures, the FNR intensities decrease for higher frequencies. The 217MHz resonance frequency corresponds to bulk face-centred-cubic (f.c.c.) Co ref. 33 in a multi-domain magnetic structure with particle sizes typically larger than 60 nm. Such large and magnetically multi-domain particles cannot become superparamagnetic at any of the FNR measurement temperatures used in this work. Therefore, the observation that the 217MHz line is not affected by the measurement temperature is in agreement with our interpretation that the losses in the FNR intensities solely arise from superparamagnetic particles that vanish from the FNR spectra with the increase of the temperature.

In the FNR spectra shown in the Fig. 1a, the 217MHz (bulk f.c.c. Co) line is the only one having a non-ambiguous origin. Indeed, for the smallest particles, for which the magnetic structure consists in a single magnetic domain, an additional magnetostatic field (the demagnetizing field, *H*_d_) is experienced by the nuclei. This magnetostatic field results in a shift of the FNR resonance lines that depends on the shape of the particles. Considering spherical particles, this shift is of the order of +6 MHz (see the Methods for details). As a consequence, if in the same sample, both multi-domain and single-domain particles coexist, two sets of lines will be superimposed (multidomain Co: 217–225 MHz refs 33, 34 and single domain Co: 223–231 MHz) making the FNR analysis very difficult to perform.

FNR spectra involve all the particles with blocking temperatures larger than the FNR measurement temperature. Therefore, in order to sample the structure of the particles for specific size ranges, we introduce the TDFNR spectra that consist in computing the difference of the spectra recorded for adjacent temperatures (2 K FNR spectrum minus 4.2 K spectrum; 4.2 K spectrum minus 77 K spectrum…). The TDFNR spectra shown in the Fig. 1b correspond to the spectra of the particles with blocking temperatures ranging between: 2 and 4.2 K, 4.2 and 77 K and above 77 K. As the blocking temperature is linked to the particle size (see the Methods for details), the differential spectra give a structural information for the particles within a specific size range of the size distribution. It is therefore possible to sample the structure of the particles for specific size ranges within their size distribution.

The TDFNR spectra present several properties: First, the integral intensity of the TDFNR spectra is proportional to the number of atoms involved in each blocking temperature range (Fig. 1c). It is similar to the derivative of thermoremanent measurements^24^. (see Supplementary Note 1 for details). Second, the TDFNR spectra obtained for the 2–4.2 K and 4.2–77 K ranges arise only from single-domain particles. Indeed, multi-domain contributions stay unchanged when the FNR spectra are measured at 2 and 4.2 K, therefore, they cancel out in the difference process. In consequence, and as expected, Fig. 1b shows that the contribution at 217 MHz (large f.c.c. multi-domain particles) is completely suppressed for these two temperature ranges (this is actually an internal test of the validity of TDFNR concept). This is also true for the higher frequency contributions. This makes the analyses of the TDFNR spectra much simpler as only one set of lines has to be considered. And finally the most important point, the TDFNR spectra for 2–4.2, 4.2–77 and >77 K allow analysing the crystallographic structure of specific size ranges of the particles within the size distribution. This information is unique and will allow a much deeper understanding of the properties of the nanoparticle distributions.

As TDFNR spectra are introduced for the first time, we propose to compare the number of particles obtained in each FNR size range with the TEM size distribution. In Fig. 1c, it is the fraction of Co atoms involved in each blocking temperature range that is represented. However, usually it is the size distribution of the particles that is given. Assuming magnetically independent spherical particles and using equation 2 in the Methods, the minimum particle sizes measurable by FNR are 1.2, 1.6 and 4.2 nm for measurement temperatures of 2, 4.2 and 77 K, respectively. However, these sizes are minimal sizes as spheres minimize the diameter of the particles for a given volume. These are therefore difficult to compare to the sizes measured by local investigation techniques like TEM. Indeed, the sizes measured by TEM strongly depend on the shape of the particles. If we consider shapes like half spheres, cubes or pancakes, their characteristic sizes can easily be twice the diameter of spheres with the same volume. In addition, these shapes are convex and do not take in consideration any corrugation. As a consequence, to take into account these effects, we suggest that a correction factor (*k*) of ∼2.5 can be applied to the minimum sizes determined for perfect spherical shapes in order to compare FNR data to the TEM data. The resulting adjusted particle sizes are therefore of the order of 3, 4 and 10 nm for measurement temperatures of 2, 4.2 and 77 K, respectively. However, these sizes are estimates only since the true parameter is the blocking temperature that depends on the volume of the particles.

Determining the number of particles (*N*_P_) involved in each temperature (size) range from the number of atoms given in Fig. 1c (equivalent to the Co mass) is achieved by considering the mass of a typical particle for each temperature range (what does not depend on the *k* factor introduced previously). For each temperature range ‘Δ*T*_*i*_', the number of particles *N*_P,Δ*Ti*_ can be computed the following way:


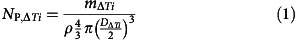


where, Δ*Ti* is the considered temperature range *i*=1 for 2–4.2 K range*, i*=2 for 4.2–77 K range and *i*=3 for >77 K range; *m*_Δ*Ti*_ is the fraction of Co mass in the temperature range Δ*T*_*i*_ (for example, for 1 mg of Co in total); *D*_Δ*Ti*_ is the diameter of the corresponding ideal spherical particle: *D*_Δ*T*1_ =1.5, *D*_Δ*T*2_=3 and *D*_Δ*T*3_=10 nm; and *ρ* is the specific mass of Co.

**Figure 2:**
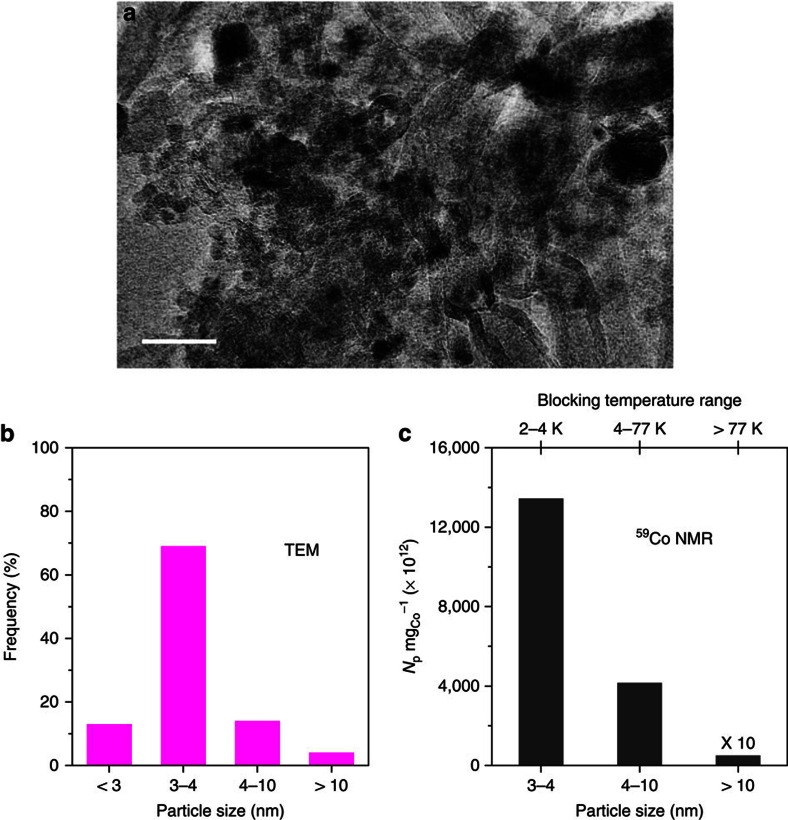
TEM and TDFNR cobalt particle size analyses. (**a**) TEM micrograph of CoCNTA sample (scale bar, 20 nm). (**b**) Corresponding cobalt particle size distribution, which is calculated from hundreds of Co particles based on the statistical TEM measurements. (**c**) Cobalt particles numbers per mg of cobalt (*N*_p_  mg_Co_^−1^) in the different particles size ranges as a function of the blocking temperature measured by TDFNR. The number of size ranges is limited to 3 because the smallest particles (<3 nm) observed by TEM cannot be measured at the lowest FNR temperature. The discrepancy observed for the large Co particles (>10 nm) between the TEM and FNR techniques could be attributed to the extremely small area of investigation. The total catalyst weight analysed by FNR is ∼100 mg while that analysed by TEM is less than a nanogram.

It must be noted that as FNR provides the atom (or mass) fraction in each blocking temperature range, it is possible to compute an absolute number of particles for each temperature range what adds to the usefulness of the information provided by our method. The number of particles obtained per mg of Co in each blocking temperature range is represented in Fig. 1d. It is compared with the TEM results. For performing this comparison, the TEM distribution has been reduced to size ranges similar to the FNR size ranges (Fig. 2). It has to be noted that although the TEM statistics has been performed on ∼200 particles, the FNR statistics is performed on typically 10^16^ particles. Therefore, the direct comparison of Fig. 2b and Fig. 2c has to be done with care. In order to limit the bias in the statistics that is due to the TEM small area of investigation (Supplementary Fig. 1), the measurements have been performed on sample CoCNTA (see next section) because it produces a large number of small particles. The bar graphs obtained by both techniques in Fig. 2 show similar trends. However, TEM analysis reveals some very small Co particles (first bar in Fig. 2b, size <3 nm) that are probably not measured by FNR because their blocking temperatures are smaller than the lowest temperature used in this work. A discrepancy can also be observed in the amount of the largest particles. The TDFNR analysis identifies a smaller number of large particles compared with the TEM results. These particles represent only rare events in the analysing area of TEM and might therefore be strongly dependent on the probed area.

However, the really original input of our work is to make possible the analysis of the structure and the chemical composition of the particles for each size range within the size distribution. This is exemplified in the two following case studies.

### Crystallographic structure sampling

**Figure 3:**
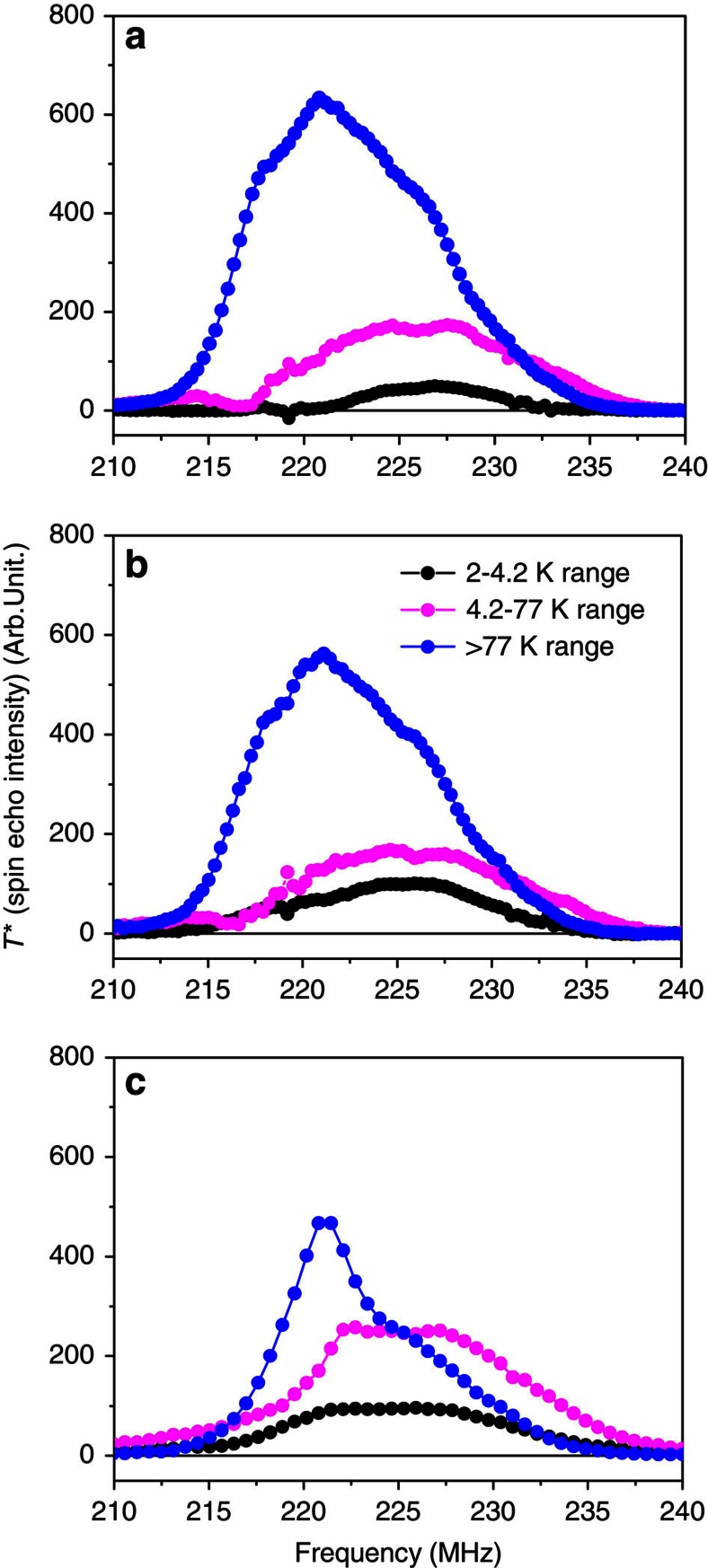
Temperature differential ferromagnetic nuclear resonance ^59^Co spectra. (**a**) CoS, (**b**) CoDTiS and (**c**) CoCNTA. Spectra (**a**,**b**) have similar shapes showing that the crystallographic structures of the samples are similar. Spectra **c** are very different from spectra **a** and **b**. The sample in **c** contains a much larger number of small f.c.c. particles (line at 222 MHz) than the samples in **a**,**b**.

The FT process that transforms synthesis gas into clean synthetic hydrocarbons involves catalytic processes in which dispersed Co nanoparticles are particularly efficient^35^,^36^,^37^. However, as the elaboration process does not allow producing mono-dispersed Co particle sizes and as Co nanoparticles can be obtained both with a hexagonal (h.c.p.) and a cubic (f.c.c.) structure^30^,^31^, the relationship between the optimum size and crystallographic structure of the cobalt active phase is still controversial. In this context, we investigated three supported catalysts by TDFNR (Fig. 3): CoCNTA^38^,^39^, CoDTiS^31^ and CoS (the sample preparations are given in the Supplementary Notes 2 and 3).

**Figure 4:**
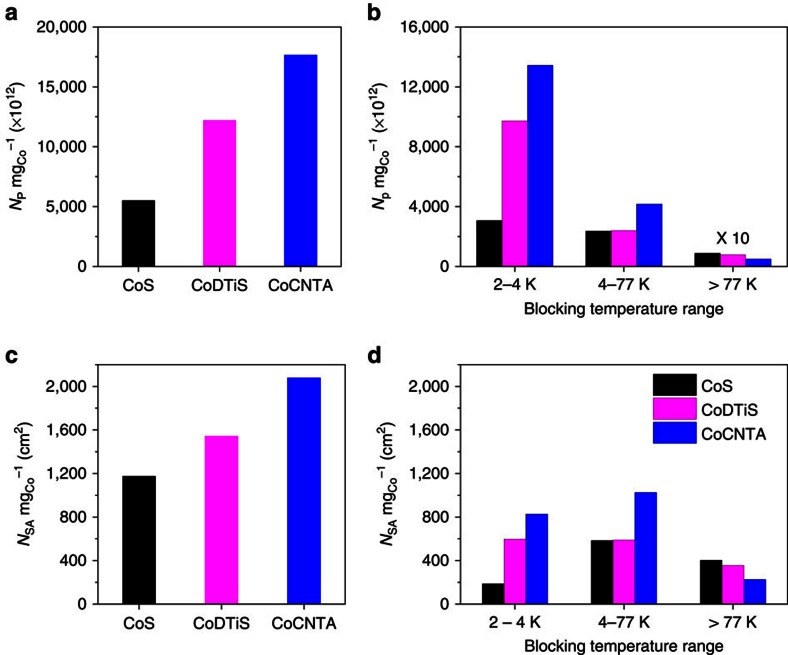
The number cobalt particles and surface area of the particles calculated from TDFNR spectra. (**a**) Total amount of metallic cobalt particles per mg of cobalt (*N*_p_  mg_Co_^−1^) produced in the 3 studied catalysts. (**b**) Number of particles involved in the blocking temperature ranges (particles size ranges) determined from the ^59^Co NMR technique. (**c**) Total cobalt particles' surface area per the mass of cobalt (*N*_SA_ mg_Co_^−1^) exposed for the FT reaction. (**d**) Particles surface area involved in each of the blocking temperature ranges (sizes).

The TDFNR spectra (Fig. 3) and the number of particles in each size range have been computed with the method described above. Figure 4a shows that the total number of particles resulting from the sample elaboration process is very different from one sample to another. Indeed, the total number of particles obtained for a given mass of Co depends on the size of the particles: If small particles are produced, the number of particles that is obtained is very large in order to accommodate a given amount of Co atoms. However, in the case of catalysis, it is the particle total surface area offered for the reaction that matters. The active particles' surface area, per mg of Co, involved in each blocking temperature range is established (Fig. 4c,d) by multiplying the number of particles in each size range by the surface area of the typical particle size involved in the corresponding temperature range. With the information provided by the TDFNR analyses, it is easy to correlate the number of particles in each size range and the corresponding structure of the Co particles with the catalytic activities of the samples (Supplementary Note 4). Table 1 shows that the activity of CoDTiS is 26% higher than the activity of CoS. The TDFNR spectra (Fig. 3a,b) of the two samples have similar shapes for all temperature ranges meaning that the Co particles in the samples have identical crystallographic structures. Therefore, the increase in activity can only be attributed to an increase in the available particle surface area. Figure 4c shows that CoDTiS exposes a surface area for the catalytic reaction that is 25% higher than for CoS, what is in perfect agreement with the increase of its catalytic activity compared with CoS. In addition, Fig. 4d demonstrates that while high and medium size particles provide similar contributions to the surface area, the surface area increase comes mostly from the particles having the lowest blocking temperature (2–4.2 K, the smallest particles). Our TDFNR analysis allows correlating the increase in the catalytic activity to the increase of the exposed surface area that is obtained through the production of very small particles.

Figure 4c,d shows that the CoCNTA catalyst exposes a surface area which is even larger than that of the two other catalysts. However, its activity is similar to the one of CoS and is smaller than the activity of CoDTiS. Figure 3 reveals that the TDFNR spectra of CoCNTA have very different shapes compared with the two other samples. The reason of its small activity has therefore to be found into the crystallographic structure of the Co particles. Indeed, for blocking temperatures larger than 77 K, a well-defined line at 222 MHz is observed. This line is attributed to f.c.c. Co in magnetic single domains (217 MHz+*H*_d_). This f.c.c. Co line is also well defined for the blocking temperate ranges of 2–4.2 and 4.2–77 K. By contrast, this line is much weaker in the TDFNR spectra of CoS and CoDTiS (especially in the temperature range of 2–4.2 and 4.2–77 K). It shows that in CoCNTA, the Co particles have a crystallographic structure containing a significant contribution of f.c.c. Co whatever the size of the particles. In CoS and CoDTiS although an f.c.c. Co contribution is also observed, it is restricted to the largest particles that do not contribute significantly to the catalytic activity. The smallest particles in CoS and CoDTiS are mostly h.c.p. Therefore, the reduced activity of CoCNTA has to be attributed to the f.c.c. structure of the smallest Co particles, which cancels the effect of the increase of the exposed surface area. Several groups have already suggested that h.c.p. Co is more active^40^,^41^; however, it was not clear if it was due to improved dispersion of the particles or to the crystallographic structure itself. In our work, we show without ambiguity that h.c.p. Co is more active by identifying independently the effect of the size of the particles (dispersion of particles) from the effect of the structure of the particles. It illustrates the effectiveness of our method. In addition, it is an important result for the community working in catalysis. A recent first-principle kinetic study seems to confirm our result^42^.

### Chemical composition and chemical ordering sampling

**Figure 5:**
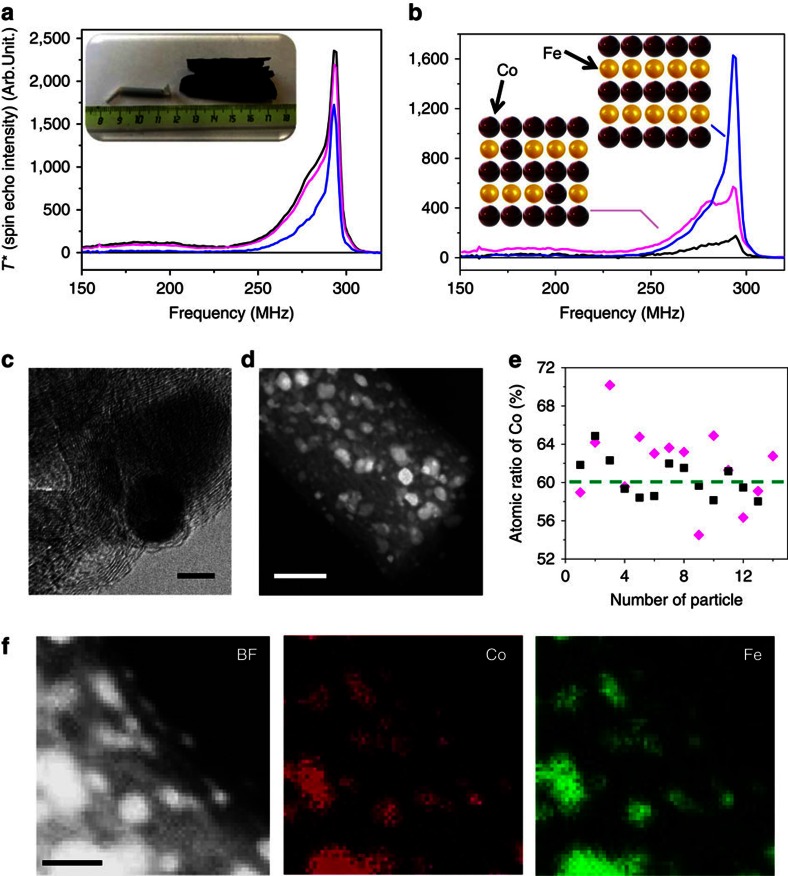
Co-Fe alloyed nanoparticles embedded in the carbon fibres. (**a**) The ^59^Co zero field FNR spectra recorded at 2 (black), 4.2 (magenta) and 77 K (blue). The inset optical photo shows the tested sample in the setup (left) and a totally expended sample (right) with the same mass (∼100 mg, with size around 3 cm by 5 cm). (**b**) TDFNR spectra: in black spectrum measured at 2 K minus spectrum measured at 4.2 K; in magenta spectrum at 4.2 K minus spectrum at 77 K; in blue spectrum at 77 K. The inset structure models display the B2 ordered Co and Fe atoms (blue spectrum) and the same ordered structure but with an excess of Co atoms (magenta and black spectra). (**c**) High-resolution TEM image of typical Co-Fe alloyed nanoparticles embedded in the porous carbon fibres. (**d**) Representative HAADF-STEM image of various size particles embedded in carbon fibres, and (**e**) relative Co and Fe atomic concentration in big (30±3 nm, black) and small (10± 3 nm, magenta) alloyed nanoparticles measured by EDS mapping. (**f**) Relative map with Co in red and Fe in green showing the Co and Fe elements dispersed in different size of alloyed nanoparticles. The scale bars in **c**,**d** and **f** are 5, 100 and 30 nm, respectively.

Our method can also be applied to the study of chemical composition and chemical order in-homogeneities within the size distribution of nanoparticles (Fig. 5). As an example, we investigated Co-Fe alloyed nanoparticles with a Co to Fe atomic ratio of 1.5 embedded in carbon fibres prepared by electrospinning followed by a thermal treatment (Fig. 5c,d,f and Supplementary Fig. 2). The Co-Fe system is often difficult to study because of the closeness of Fe and Co in the periodic table and constitutes therefore an interesting test system for our method. The comparison of the size distributions obtained by TDFNR and by TEM is given in the Supplementary Fig. 3. The TDFNR spectra (Fig. 5b) show a main line at 290 MHz, which is the fingerprint of Co atoms in a B2 ordered Co-Fe alloy^43^. The B2 structure is constituted by alternate atomic planes of Co and Fe atoms. A perfectly ordered and stoichiometric Co-Fe alloy would result in a single NMR line close to 290 MHz (ref. 43). The observation that, for low blocking temperatures, the intensity at 290 MHz decreases (accompanied by an increase of low frequency contributions), is attributed to the presence of an excess of Co atoms in the B2 phase. By comparison with Jay *et al*.^43^, we can propose that the largest particles have a Co content of the order of ∼55%, while the smallest particles have a Co content close to 65%.

The chemical compositions measured by TEM (Fig. 5e, Supplementary Fig. 4 and Supplementary Note 5) are rather scattered. However, the smallest particles seem to show also a larger Co concentration. TEM data do not fully confirm TDFNR results but do indicate a tendency that is in agreement with TDFNR. In this example, TDFNR provides a very unique insight into the samples morphology by simultaneously sampling the chemical composition and chemical order versus the size of the Co-Fe nanoparticles.

## Discussion

In this work, we have shown that through TDFNR spectra we can sample the crystallographic structure, chemical order and chemical composition of non-interacting ferromagnetic nanoparticles for specific size ranges within their size distributions. To our knowledge, no other technique allows obtaining simultaneously such information on large-scale samples. The methodology has been applied to the study of dispersed Co nanoparticles for catalysis and we have demonstrated that the presence of f.c.c. Co strongly reduces the catalytic activity even if the size of the particles is reduced in order to maximize the exposed surface area for the reaction. The TDFNR spectra were also successfully used to sample simultaneously the chemical composition and chemical order within the size distribution of alloyed Co-Fe nanoparticles. In the field of catalysis, this can be efficiently used to determine the influence of promoters on the main active phase by visualizing the direct incorporation of these promoters inside the structure of the ferromagnetic phase (that is, cobalt phase promoted with noble metals). The method has been demonstrated for Co-based samples but can be applied to many ferromagnetic nanoparticles since most elements have NMR active isotopes (Ar and Ce being the only elements having no isotopes with non-zero nuclear spin). Therefore, our methodology can be applied to investigate the relationship between the properties the structure and chemical composition of non-interacting ferromagnetic nanoparticle distributions in many research fields such as spintronics, ferro fluidics, biology or medicine.

## Methods

### NMR in ferromagnets

As NMR in ferromagnets (FNR) is not well known, we remind here the specificities of NMR when used for studying ferromagnets^2^,^3^. The first specific feature is that the application of an external static magnetic field used to lift the nuclear spin degeneracy (usually called *H*_0_) is not needed. Indeed, in ferromagnets, the nuclei already experience a static magnetic field originating from the magnetization of the sample that is called the hyperfine field (HF). In bulk Co, the HF is typically of the order of 20 Tesla. Therefore, we usually perform NMR measurements in ferromagnets by frequency sweeping without applying any external static field. That is why it is also called zero-field NMR or internal field NMR. The second particularity is that the radiofrequency field usually called *H1* is not directly experienced by the nuclei. The radiofrequency field *H*_1_* experienced by the nuclei is a field enhanced by the local magnetic susceptibility of the sample. This is systematically taken into account when establishing the FNR spectra. Indeed, the FNR spectra are recorded for at least five different values of the excitation radio frequency (RF) field power, covering a range over more than one order of magnitude. This procedure allows determining the optimum excitation field power at each frequency and allows taking into account the variation of the local electronic susceptibility as a function of frequency^2^,^3^. After this, a further correction for the regular frequency dependence of the NMR signal is applied. The FNR amplitudes obtained in such a way represent the true distribution of atoms (nuclei) with the HF. This method has been implemented in our group and became a standard procedure.

**Figure 6:**
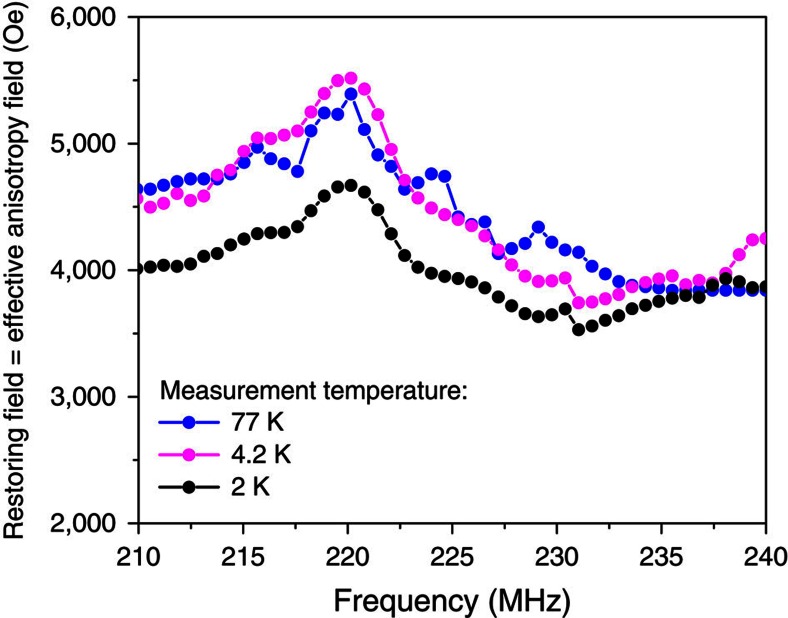
Example of effective anisotropy field obtained from the measurement of the FNR spectra. Effective anisotropy measured at 77 (black), 4.2 (magenta) and 2 K (blue). No significant variation of the effective anisotropy can be observed with the temperature.

The local electronic susceptibility measured by FNR is converted into a restoring field^2^,^3^ that in the case of the nanoparticles under investigation can be identified to their effective anisotropy field. This allows measuring the magnetic anisotropy of the system directly from the FNR data. Examples of effective anisotropy fields obtained for different measurement temperatures are shown in Fig. 6. This plot shows that whatever the Co structure the anisotropy is similar for all structures (it is fairly flat with frequency), that the average value is close to the bulk value of Co (4 × 10^6^ erg cm^−3^, whereas for h.c.p. Co it is 5 × 10^6^ erg cm^−3^), and that the anisotropy is similar whatever the measurement temperature.

The ^59^Co zero field NMR experiments were performed in a home-made spectrometer. The integrated spin-echo intensity was recorded using a broadband un-tuned (high-pass design) pulsed NMR spectrometer with phase-sensitive detection and automated frequency scanning (pulse length: 5 μs, delay: 3 μs). The frequency response of the setup is flat within 0.5 dB. The FNR measurements were performed at 2, 4.2 and 77 K in order to be able to obtain information about crystal microstructure, chemical composition and size distribution of Co-based particles in the samples. In order to take into account the regular 1/*T* dependence of the FNR intensities, all the spectra intensity have been multiplied by their respective measurement temperature.

### Demagnetizing fields in single-domain particles

In bulk cobalt, the FNR spectra show well known resonance frequencies at 4.2 K: 217 MHz for f.c.c. Co and from 220 to 225 MHz for h.c.p. Co and stacking faults. These reference frequencies are valid only for macroscopic ferromagnetic samples with a so-called multi-domain magnetic structure. When the size of the sample becomes smaller, it is energetically not favourable anymore for the object to be in a multi-domain magnetic structure and the object becomes single domain. When the magnetization in a small object points in one single direction, it is called magnetically single domain. This single-domain magnetic structure results in stray fields outside of the sample and in demagnetizing field inside the sample. As the nuclei experience the magnetic field inside the sample, they will also experience the demagnetizing field. The demagnetizing field depends on the shape of the sample but for a sphere it is of the order of 0.6 Tesla resulting in a FNR line shift of +6 MHz. The frequency shift is positive because the HF in metallic Co is negative with respect to the magnetization direction and is therefore in the same direction as the demagnetizing field.

### Volume and blocking temperature relationship in nanoparticles

For non-interacting objects, the particle volume and the particle blocking temperature are linked through the following formula^32^:


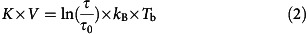


where *K* is the anisotropy constant of the ferromagnetic material (5 × 10^6^ erg cm^−3^ for Co; 3 × 10^5^ erg cm^−3^ for CoFe), *V* is volume of the particle, *τ* is the characteristic time of measurement (10 microseconds in our NMR set up), *τ*_0_ is the test time for a particle to turn back (between 10^−9^ to 10^−10^ s), *k*_B_ is the Boltzmann constant (1.38 × 10^−16^ Erg·Kelvin^−1^) and *T*_b_ is the blocking temperature in Kelvin, respectively.

The use of this formula assumes that the Co particles are magnetically independent. In the samples under investigation, we consider that it is a good approximation for the following reasons. At first, the Co particles represent 10% of the total mass of the sample and if we consider the densities of the Co and of the support the volume fraction of the Co particles is only of the order of 3%. In addition for sake of catalysis efficiency, the sample preparation is optimized in order to disperse as much as possible the particles on the support. Second as shown in Fig. 6, FNR also measures simultaneously the effective magnetic anisotropy of the sample. Magnetic coupling between the particles would result in a strong decrease of the samples anisotropy when the measurement temperature is decreased^44^,^45^. This is not observed in Fig. 6, the magnetic anisotropy of the samples is similar to the one of bulk Co and does not vary significantly with temperature.

### TEM size distributions

The size distribution histogram of each catalyst was counted based on more than 200 particles taken from different micrographs. For improving the statistics, the measurements were carried out with several sets of images corresponding to different regions. Data acquisition was performed by using DigitalMicrograph software and the histograms were built up after manual measurements of the particle sizes.

## Additional information

**How to cite this article:** Liu, Y. *et al*. Sampling the structure and chemical order in assemblies of ferromagnetic nanoparticles by nuclear magnetic resonance. *Nat. Commun.* 7:11532 doi: 10.1038/ncomms11532 (2016).

## Supplementary Material

Supplementary Information Supplementary Figures 1-4 and Supplementary Notes 1-5

## Figures and Tables

**Table 1:** Comparison of the FT catalytic performance of various cobalt-based catalysts*.

**Sample**	**CO conversion (%)**	**C**_**5+**_ **selectivity (%)**	**CoTY**†
			
CoS	27	95	4.0
CoDTiS	34	95	5.0
CoCNTA	28	92	4.2

CoTY, cobalt time yield; FT, Fischer–Tropsch.

^*^All data were obtained after 20 h of time on stream where stable catalytic performance at testing conditions was achieved. Reaction conditions: reaction temperature=215 ^o^C, gas hourly space velocity (GHSV) standard temperature and pressure (STP)=3,600 ml g_cat_^−1^ h^−1^, H_2_/CO=2, pure syngas, total pressure=40 bar. The catalysts tested have already been evaluated in the FT reaction at 215 ^o^C about 3 days.

^†^Cobalt time yield (10^−5^ mol_co_ g_Co_^−1^ s^−1^, molar CO conversion rate per gram of Co per hour).
