# Supplementary material for: Sampling the structure and chemical order in assemblies of ferromagnetic nanoparticles by nuclear magnetic resonance
Source: Nat Commun. 2016 May 9;7:11532. doi: 10.1038/ncomms11532 (PMC4865821; doi:10.1038/ncomms11532)
Supplement: Supplementary Information — Supplementary Figures 1-4 and Supplementary Notes 1-5 [file ncomms11532-s1.pdf]

Supplementary Figure 1

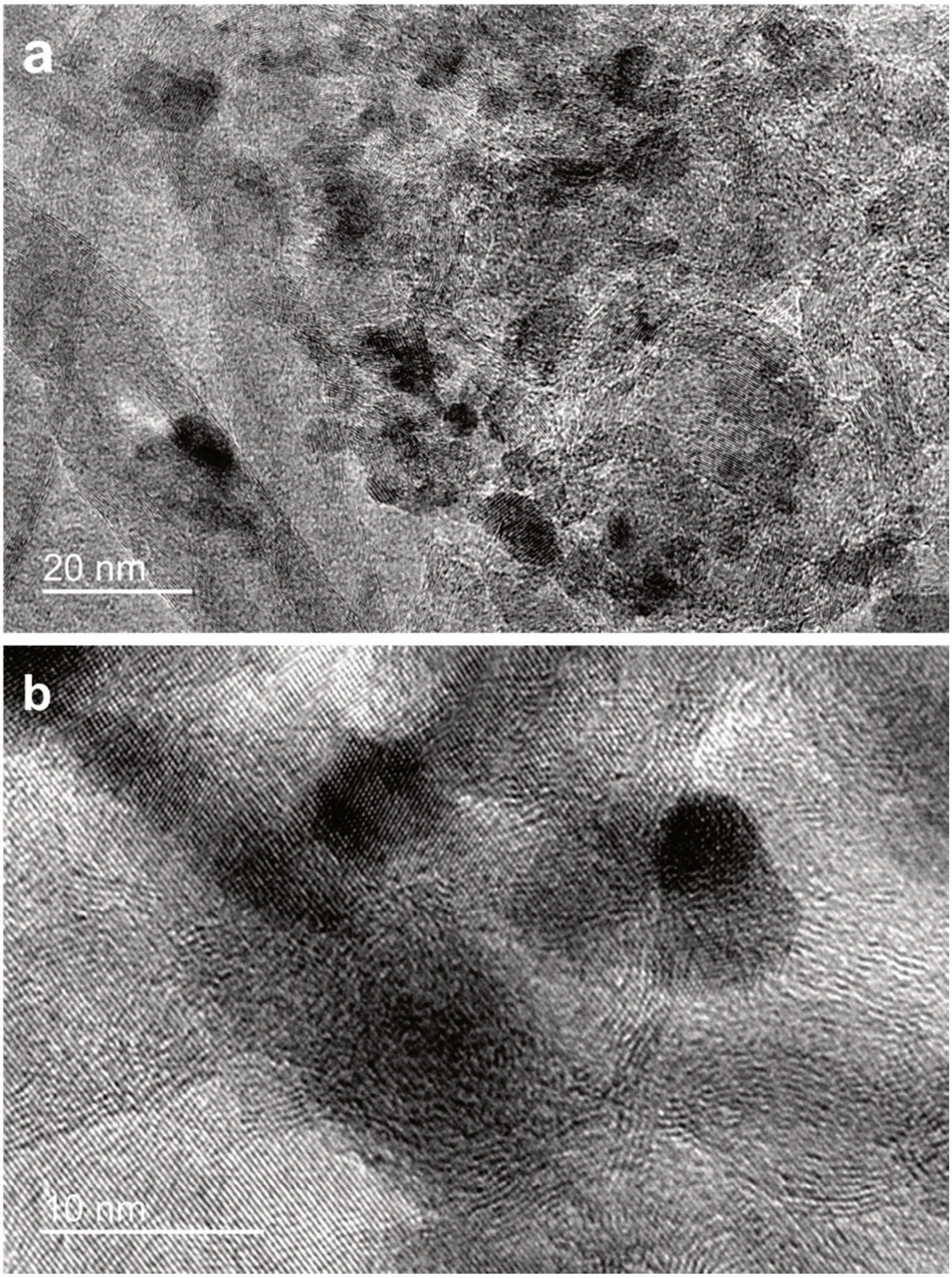

Representative TEM and HRTEM micrographs. The sample CoCNTA shows the presence of small cobalt particles (darker parts in the pictures).

Supplementary Figure 2

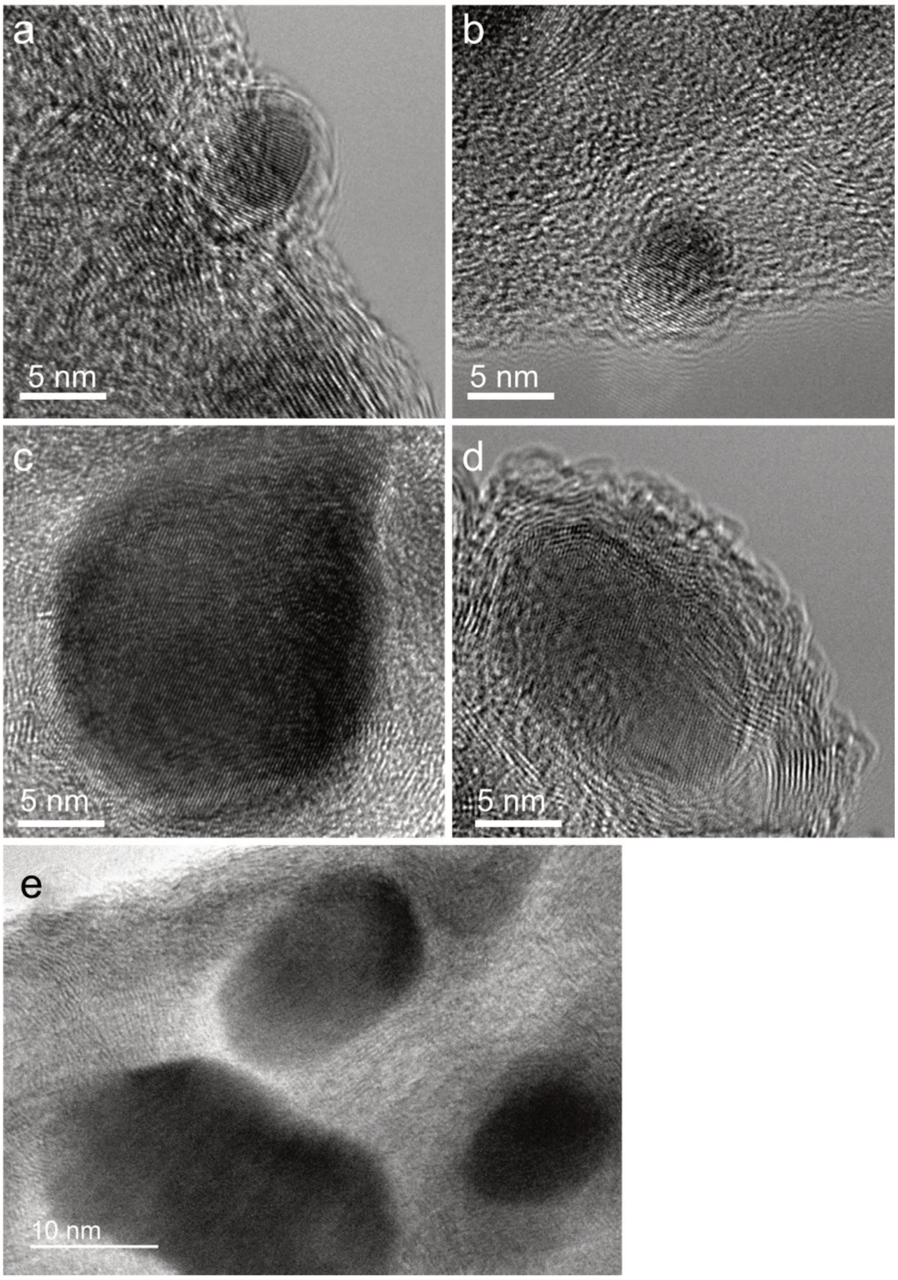

Typical HR-TEM image of Co-Fe alloyed nanoparticles sample. Images show small as well as big particles. No contrast can be observed between Fe and Co.

Supplementary Figure 3

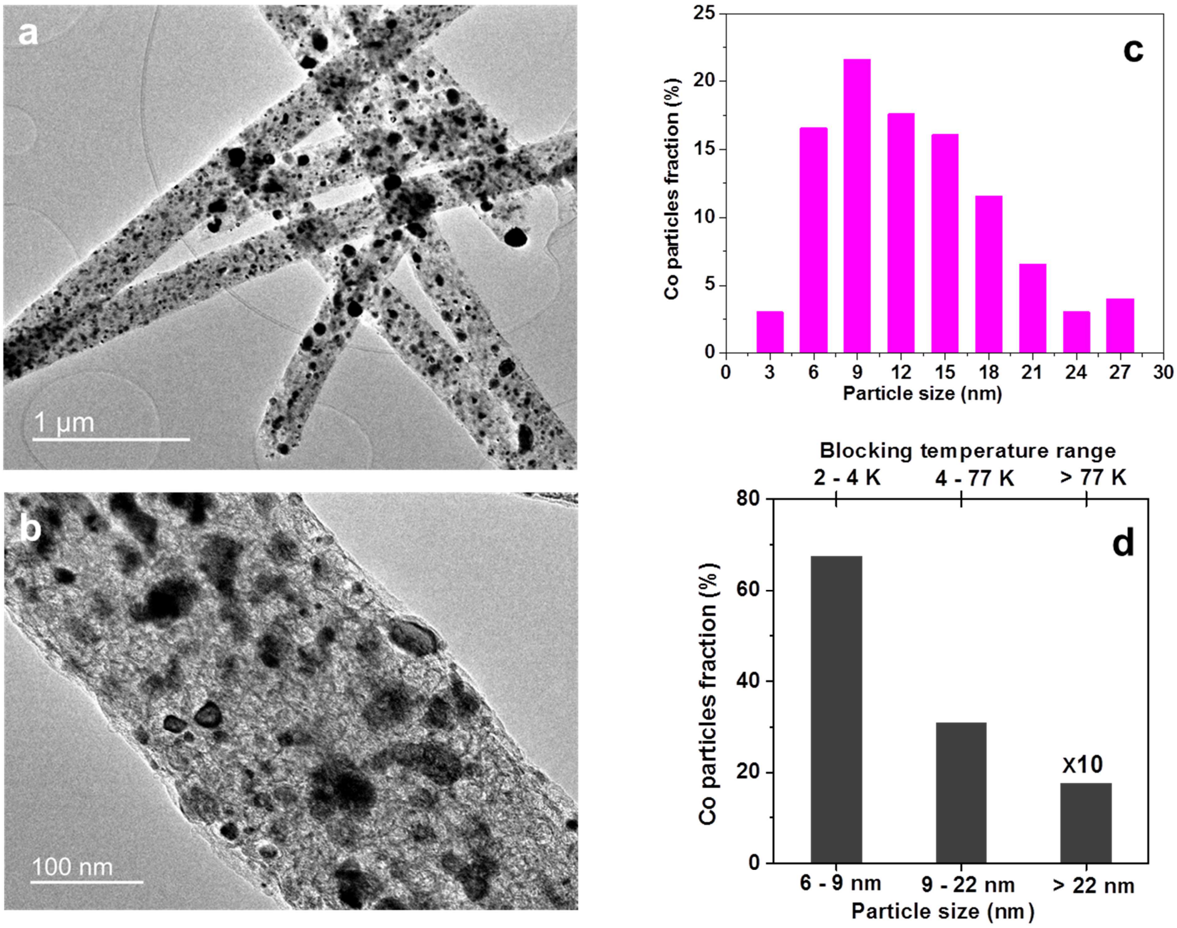

TEM analyses of CoFe nanoparticles. (a, b) Representative TEM micrographs of CoFe@CF sample. (c) Corresponding cobalt particle size distribution which is calculated from hundreds of Co particles based on the statistical TEM measurements. (d) The cobalt particles distribution in the sample as a function of the blocking temperature measured by ^{59}Co NMR (the size ranges corresponding to the blocking temperature ranges have been adjusted to take into account the smaller magnetic anisotropy of CoFe alloys compared to bulk Co; equations 1 and 2).

Supplementary Figure 4

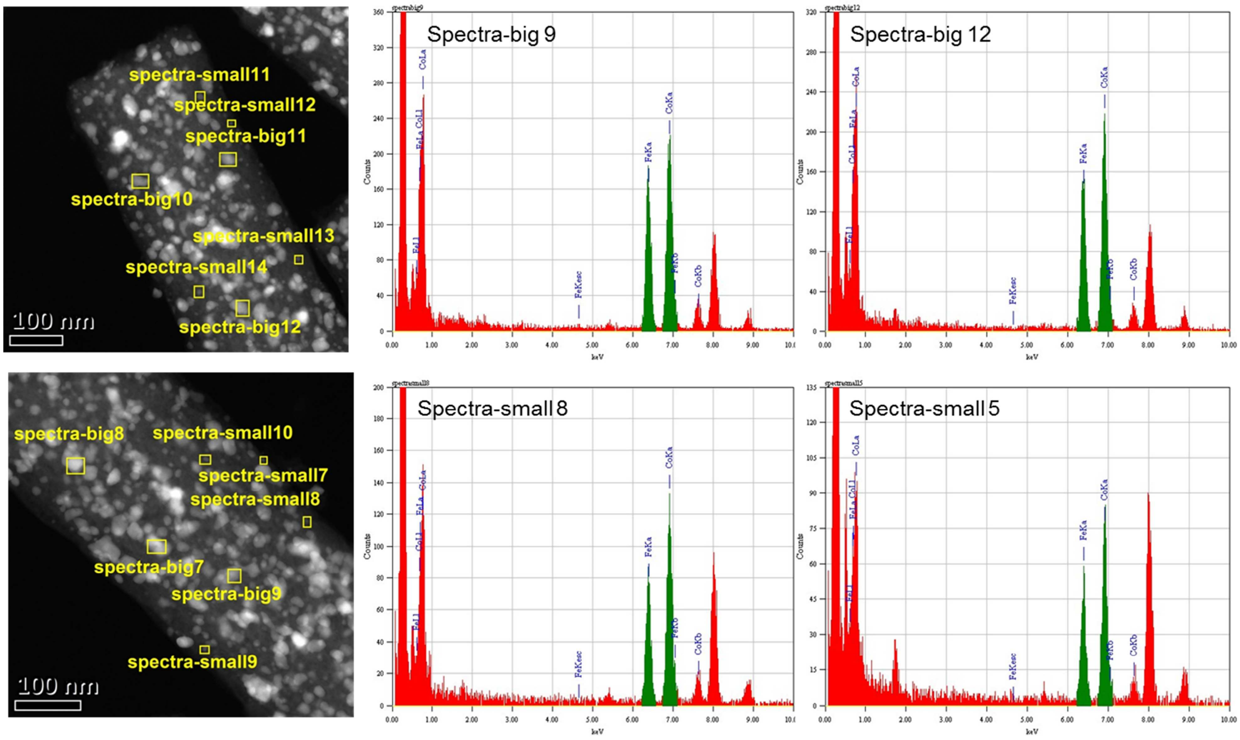

Chemical analyses of the CoFe particles. HAADF-STEM and EDS maps of big (30 ± nm) and small (10 ± nm) cobalt-iron alloyed nanoparticles.

Supplementary Note 1

Relationship between TDFNR integral intensity and blocking temperature distribution:

If $N(T_b)$ is the number of atoms having a T_b blocking temperature, the number of atoms measured by FNR ($FNR(T)$) at a given temperature T is the sum of all the atoms with $T_b>T$:

$$FNR(T) = \int_T^{\infty} N(T_b)\mathrm{d}(T_b) \qquad (1)$$

Supplementary equation 1 is formally identical to equation 5 in *(45)*. Therefore $N(T_b)$ can be identified to the distribution function of blocking temperatures.

When computing TDFNR intensities between T_1 and T_2

$$FNR(T_1) - FNR(T_2) = \int_{T_1}^{\infty} N(T_b)d(T_b) - \int_{T_2}^{\infty} N(T_b)d(T_b) = \int_{T_1}^{T_2} N(T_b)d(T_b) \qquad (2)$$

$FNR(T_1)$-$FNR(T_2)$ is therefore an approximate of $N(T_b)$ and an approximate of the distribution function of blocking temperatures.

Supplementary Note 2

Preparation of supported Co nanoparticles

Cobalt supported catalysts with a cobalt loading of 10 wt. % were synthesized by pore volume impregnation method using an ethanol-water (50:50 v:v) solution containing cobalt nitrate. The as-prepared materials (typically 5 g), were impregnated by 7 mL of ethanol-water solution to which 2.74 g $Co(NO_3)_2\cdot 6H_2O$ was added. After impregnation, the solid was allowed to dry at room temperature for 2 h and then oven-dried at 110 °C for 8 h. The solid was calcined in air at 300 °C for 2 h in order to decompose the nitrate into its corresponding oxide. The oxide form

was further reduced in flowing hydrogen (30 $mL·g_{cat}^{-1}·min^{-1}$). In order to prevent excessive air oxidation during the exposure of the catalyst, a passivation process was performed before discharging of the catalyst (surface passivation was carried out with a mixture of 1 vol. % O_2 diluted in helium at room temperature). The cobalt was loaded on the thermal conductive support materials such as macroporous β-SiC (40 $m^2·g^{-1}$), TiO_2 doped β-SiC (101 $m^2·g^{-1}$) and CNT-α-Al_2O_3 (76 $m^2·g^{-1}$), and the obtained catalysts were named CoS, CoDTiS and CoCNTA, respectively. This case study was performed on spent catalysts covered with a homogeneous solid wax layer in order to prevent any surface oxidation of the cobalt nanoparticles during air exposure.

Supplementary Note 3

Preparation of Co/Fe nanoparticles embedded in carbon fibers (Co/Fe@CF) by electrospinning process

0.5 g polyacrylonitrile (PAN, Mw = 200 $kg.mol^{-1}$) and 0.5g polyvinylpyrrolidone (PVP, Mw = 1300 kg.mol-1, Aldrich) were dissolved in 9g of dimethylformamide (DMF) at 50 °C under vigorous stirring for 10 h. After cooled down to room temperature, 0.25 g cobalt(II) acetate tetrahydrate ($Co(ac)_2·4H_2O$, ⩾ 98%, Aldrich) and 0.25 g iron(III) acetylacetonate ($Fe(C_5H_7O_2)_3$, > 97%, Aldrich) were then added and the mixture was vigorously stirred for 12 h before used as the precursor solution for electrospinning. A parchment paper covered collector was attached on a rotating mandrel to collect the as-spun fibers. A rotation speed of 100 rpm of the mandrel was applied to obtain fibrous sheets with homogeneous thickness. The precursor solution was delivered with a constant flow rate of 1.2 $mL·h^{-1}$ using a syringe pumps (Fischer

scientific). The distance between the needle and the collector was 15 cm. A positive potential Vneedle = 12 kV was applied on the needle and a negative potential Vcollector = - 4 kV was applied on the collector using power supplies (Spellman SL10). The as-collected electrospun fibers were first stabilized in air at 250 °C for 2 h with a heating rate of 1 $°C\cdot min^{-1}$ and subsequently carbonized at 800 °C for 3 h with a heating rate of 5 $°C\cdot min^{-1}$ in argon atmosphere to finally obtain the Co/Fe@CF. The as-prepared samples were further reduced in flowing hydrogen at 300 °C for 2 h.

Supplementary Note 4

Fischer - Tropsch synthesis

The Fischer-Tropsch synthesis was performed at 40 bar in a tubular fixed-bed stainless steel reactor (inner diameter = 6 mm) with circulating silicon oil as heating source. Typically, reduced catalysts (the macroscopic size ranged between 0.15 to 0.40 mm) were deposited between quartz wool plugs in the middle of the reactor. The reactor pressure was slowly increased from 1 to 40 bar (ramping rate of 10 $bar\cdot h^{-1}$) under argon via a back pressure regulator (MFI Ltd). At 40 bar, the reactor temperature was raised from room temperature to the initial reaction temperature (215°C), with the heating rate of 2°C·min^{-1}. Then, the argon flow was replaced by a 50:50 v:v mixture of synthesis gas (syngas, H_2/CO molar ratio of 2) and argon. The catalyst was activated under a syngas-argon mixture with different syngas concentrations before being evaluated under pure syngas conditions. The catalyst bed temperature was monitored with a thermocouple (Ø 0.3 mm) inserted inside a stainless steel finger (Ø 1 mm) passing through the catalyst bed. The products were condensed in two high pressure traps maintained at 85°C and 15°C, respectively. The outlet gases (C_1-C_6 hydrocarbons, and CO_2 as well as H_2 and unconverted CO) were

analyzed on-line by gas chromatography (GC), both by Thermal Conductivity Detector (TCD) and Flame Ionization Detector (FID).

Supplementary Note 5

TEM-based analyses

For (S)TEM analysis, several droplets of powdered material suspended in acetone by sonication were deposited onto a cooper grid covered by a holey carbon membrane. Conventional and scanning transmission electron microscopy analyse were performed on a JEOL 2100 F (S)TEM electron microscope operating at 200 kV and equipped with a Cs corrected condenser allowing to reach a resolution of 0.11 nm in STEM. The HAADF-STEM mode is based on scanning a sample by a focused electron probe and detecting high-angle scattered electrons using an annular dark-field detector (HAADF) In this mode, the proportionality between the intensity and nuclear charge is typically equal to $Z^{1.8}$ which allows obtaining a good contrast in the corresponding images, in order to investigate elements with large difference between their atomic number, such as metallic nanoparticles deposited on a lighter element support. The camera length used in HAADF mode was 10 cm, corresponding to inner and outer diameters of the annular detector of 60 mrad and 160 mrad.
